# HIV Prevention and Treatment Fields Join Forces

**DOI:** 10.1016/j.ebiom.2014.10.009

**Published:** 2014-10-24

**Authors:** Steven G. Deeks, Robert Siliciano

**Affiliations:** a University of California, San Francisco, United States; b Johns Hopkins University School of Medicine, Howard Hughes Medical Institute, Baltimore MD, United States

There remains no doubt that the development of antiretroviral therapy (ART) for the prevention and treatment of HIV infection has been one of the greatest medical advancements in the past few decades. When those without HIV use ART appropriately as either pre-exposure prophylaxis (PrEP) or post-exposure prophylaxis (PEP), HIV acquisition is almost universally prevented (Grant et al., 2010). Similarly, when those with HIV use therapy appropriately, transmission to others rarely if ever occurs (Cohen et al., 2011), and the risk of developing AIDS is completely eliminated.

There are over 35 million people living with HIV, and many millions more at risk for acquisition. Most live in resource poor regions. Delivering these expensive drugs to those in need, and getting people to adhere to complex regimens indefinitely, is a daunting challenge. Without unprecedented investments in global health and implementation science, it is unclear if ART alone will ultimately eliminate the epidemic.

The HIV research agenda has therefore shifted. The main biomedical goals for discovery and translational science are to develop an effective vaccine for those at risk for HIV and to develop a safe, scalable, short-term intervention that results in durable remission for those with HIV (in others words, a “cure”). Although the prevention and treatment research agendas are often competing for dwindling research dollars, the reality is that in the past few years these agendas have begun to merge. The distinction between prevention and a cure is now surprisingly hard to define, and many of the most exciting advances in vaccine research are now being actively repurposed for cure research.

We and others have commented on the PreP-PEP-cure continuum (Barouch & Deeks, 2014). When HIV infects activated CD4 + T cells, HIV DNA encoding replication-competent virus is successfully integrated into the host cell genome. Most of these cells die rapidly, but rare cells may survive and return to a resting state that is non-permissive for HIV gene expression. These cells persist as long lived memory CD4 + T cells (the “latent reservoir”), preventing cure by ART (Siliciano et al., 2003). If ART after infection blocks the establishment of a latent reservoir, and the infection eventually cleared, is that PEP or a cure? This is more than a theoretical debate, as is illustrated by the well-publicized “Mississippi Baby” case (Persaud et al., 2013). ART given at hour 31 of life to an infant who was clearly infected nearly prevented establishment of a latent reservoir, as evidence by the fact she had no viral rebound 18 months later when therapy was stopped. Unfortunately, this prevention was not complete, as the child eventually exhibited viral rebound after more than two years off therapy. Nevertheless, this case encouraged those in the field to pursue other studies in which very aggressive ART during acute infection will be used to prevent the establishment of latent infection.

Decades of investment in HIV vaccine efforts have led to a number of breakthroughs that are reshaping the prevention and cure agendas. Louis Picker and his team have repurposed cytomegalovirus (CMV) as a live vector to deliver SIV (and soon HIV) peptides to the immune system, resulting in the durable generation of polyclonal virus-specific CD8 + effector memory T cells. Although this vaccine does not prevent acquisition of SIV in macaques, some infected animals clearly eliminate infection soon after acquisition (Hansen et al., 2013). One likely explanation is that the pre-existing vaccine induced CD8 + T cell response eliminated infected cells before they could revert back to a resting state and establish latency. These experiments—which as of now have been limited to non-human primate models—provide the first definitive proof that established SIV (and, by extension—HIV) infection may be curable. Although the first series of studies in humans will likely focus on the role of CMV vector-based vaccines as prevention for HIV-uninfected individuals, it is expected that this approach will rapidly be studied in a curative setting.

The encouraging results from the RV144 vaccine study suggest that envelope-specific antibodies can prevent HIV acquisition (Haynes et al., 2012). Detailed studies of those with HIV have led to the identification of a growing number of antibodies that are potent and broadly neutralizing. While the prevention field is struggling with the daunting challenge of identifying an immunogen strategy to generate these complex antibodies in uninfected persons, those interested in cure are rapidly repurposing these broadly neutralizing antibodies as potential therapies. Theoretically, such antibodies can be engineered to recognize and clear infected cells perhaps by stimulating antibody-dependent cell-mediated cytotoxicity (Barouch et al., 2013). This approach, when preceded by interventions that reactivate latent HIV, could be an important addition to cure strategies, particularly since existing CTL responses in patients on ART are unlikely to be sufficient (Shan et al., 2012).

Funds for biomedical research are plummeting. This can force disciplines to compete aggressively for those funds, or find ways to work together. The natural overlap between prevention and treatment agendas in the HIV arena has led to a number of highly productive cross-disciplinary collaborations, and a palpable sense that research in the future will be more collaborative. Major investments into team science and the development of large, multi-institute “collaboratories” that encourage experts from prevention and treatment to work together could go a long way to addressing some of the most vexing problems that ART will never be able to address.

We declare that we have no conflicts of interest.
